# Analysis of millimetre-wave polarization diverse multiple-input multiple-output capacity

**DOI:** 10.1098/rsos.150322

**Published:** 2015-12-23

**Authors:** Nicholas P. Lawrence, Brian W.-H. Ng, Hedley J. Hansen, Derek Abbott

**Affiliations:** 1 School of Electrical and Electronic Engineering, The University of Adelaide, South Australia 5005, Australia; 2 RFT Group EWRD, DSTO, PO Box 1500, Edinburgh, South Australia 5111, Australia

**Keywords:** microwaves, polarization, diversity

## Abstract

Millimetre-waves offer the possibility of wide bandwidth and consequently high data rate for wireless communications. For both uni- and dual-polarized systems, signals sent over a link may suffer severe degradation due to antenna misalignment. Orientation robustness may be enhanced by the use of mutual orthogonality in three dimensions. Multiple-input multiple-output polarization diversity offers a way of improving signal reception without the limitations associated with spatial diversity. Scattering effects often assist propagation through multipath. However, high path loss at millimetre-wave frequencies may limit any reception enhancement through scattering. We show that the inclusion of a third orthogonal dipole provides orientation robustness in this setting, as well as in a rich scattering environment, by means of a Rician fading channel model covering all orientations for a millimetre-wave, tri-orthogonal, half-wave dipole transmitter and receiver employing polarization diversity. Our simulation extends the analysis into three dimensions, fully exploiting individual sub-channel paths. In both the presence and absence of multipath effects, capacity is observed to be higher than that of a dual-polarized system over the majority of a field of view.

## 1. Introduction

Consumer wireless applications are driving demand for increased user capacity, reliability and throughput. Performance should ideally be consistent regardless of end user position and orientation. Multiple-input multiple-output (MIMO) signalling techniques exploiting spatial diversity through channel scattering have been widely adopted in wireless terrestrial applications to increase performance [1–3]. Currently employed systems use uni- or dual-polarized propagation due to their ease of implementation. From a simple geometrical analysis, performance is seen to be reliant on relative antenna positions as these types of polarization do not account for a three-dimensional environment. Orientation robustness becomes an important limiting factor, as design frequency increases to cope with higher data rates, as constant linear transmit power becomes typically harder to maintain and the benefits of multipath effects at the receiver are reduced, the consequence of which is suboptimal performance, or an exponential rise in system cost to correct this. Every possible design advantage needs to be sought.

Implementation at the widely adopted mobile communication frequencies in the low microwave region typically benefits from a scattering environment but finds itself restricted to progressively complex processing techniques if it is to keep up with consumer demand for higher capacity. In the infrared region, propagation limitations are well documented [4]. As a result, much interest has been given to the terahertz region where high data rate, together with an unallocated portion of spectrum, opens the door to many possibilities [5]. Implementation at terahertz frequencies, in contrast to that at microwave frequencies, has been limited by available transmit power leading to a line of sight (LoS) system design, devoid of scattering mechanisms for increasing received energy at the receiver. In order to avoid link failure when LoS propagation is interrupted, steerable dielectric mirrors have been introduced [6].

Innovative design at millimetre-wave frequencies offers many of the advantages of both microwave and terahertz frequencies while minimizing the disadvantages [7]. First, a large 7 GHz frequency band at 60 GHz, within the millimetre-wave spectrum, has been allocated to wireless design over short distances [8]. This supports high data rate using low order modulation techniques, such as binary phase shift keying. Second, any design and implementation may be influenced by well-documented microwave techniques. Third, available power at this frequency does not necessarily restrict the system to LoS propagation. As a result, simple omnidirectional antenna configurations may be employed at the transmitter to improve performance through diversity [9]. Signal propagation may be further enhanced by the propagation environment itself, although this enhancement is typically lower at these frequencies than at lower microwave frequencies.

Dual polarization has been seen to enhance capacity in a LoS communication environment where relative transmitter–receiver antenna alignment varies slightly with relative position [10]. Spherical geometry required in satellite communication design is useful for demonstrating the benefit of polarization in three dimensions.

**Figure 1.**
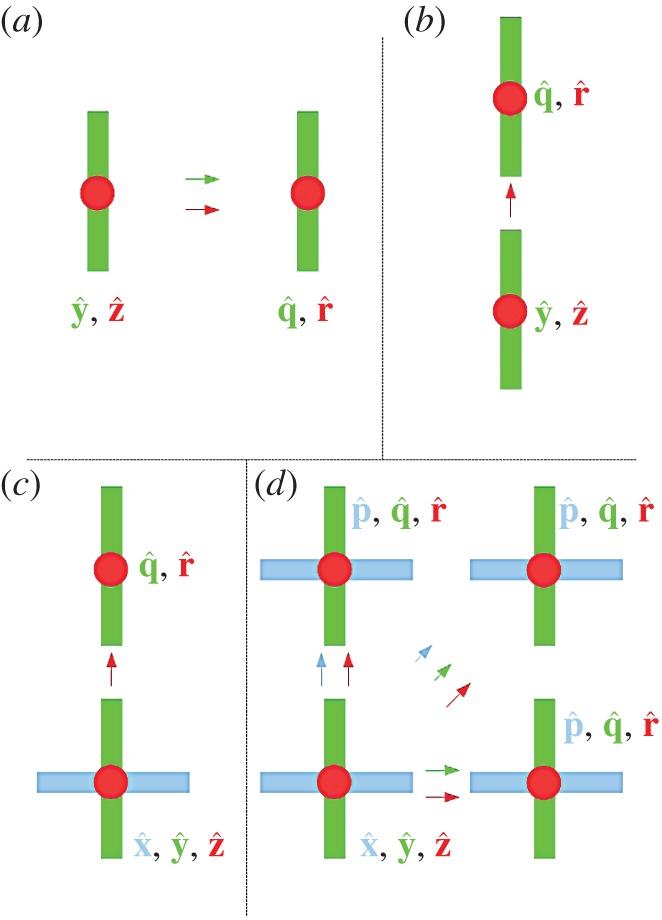
Tri-orthogonal arrangement: (*a*) full capacity is observed between receiver consisting of orthogonal dipoles $\hat{\text{q}}$, $\hat{\text{r}}$, and transmitter consisting of dipoles $\hat{\text{y}}$, $\hat{\text{z}}$, as a result of perfect alignment. Dipoles $\hat{\text{z}}$ and $\hat{\text{r}}$ are vertical so point upwards out of the paper; (*b*) half capacity is observed at the receiver as only dipole $\hat{\text{r}}$ is broadside to the transmitter; (*c*) dipole $\hat{\text{x}}$ is introduced at the transmitter. Half capacity is once again observed as only dipole $\hat{\text{r}}$ is broadside to the transmitter; (*d*) full capacity is restored through inclusion of dipole $\hat{\text{p}}$ at the receiver. At least two orthogonal polarizations are offered in any link direction.

Inclusion of a third orthogonal dipole at the antenna, leading to a tri-orthogonal configuration, enhances performance beyond that of a dual-polarized system by mitigating antenna misalignment [11,12]. This is demonstrated in figure 1.

Nine links, or subchannels, are provided in figure 1*d* by three orthogonal dipoles, denoted as $\hat{\text{x}}$, $\hat{\text{y}}$ and $\hat{\text{z}}$ at the transmitter and at the receiver denoted as $\hat{\text{p}}$, $\hat{\text{q}}$ and $\hat{\text{r}}$. Three transmitted signals, one from each of the transmitter dipoles, may be received by each of three dipoles at the receiver. Theoretically, capacity is maintained in any given direction as, due to symmetry in six principal directions as a tri-orthogonal approach is assumed, dual polarization is offered over all unit vector directions. In the instance of LoS propagation, polarization diversity offers the benefit of MIMO signalling techniques that is not always the case for a spatially diverse system.

The benefit of a rich scattering environment may further enhance this performance [2] and is often cited in reference papers pertaining to MIMO systems [11–13]. Such an environment is not typically available at millimetre-wave frequencies. An arrangement of three dipoles, at the antenna, may provide yet greater capacity if the orthogonality criteria between dipoles are relaxed [14]. However, this arrangement does not optimize capacity for all propagation directions in a field of view (FoV) in which all antenna orientations are observed. A wireless channel is a time-varying combination of a LoS signal together with a non-line of sight (NLoS) component arising from the channel environment and multipath. Relative transmitter–receiver motion may be introduced. Due to orthogonality, the electric field orientations of the propagating electromagnetic signals are affected independently, reducing correlation which, together with any variation in the channel environment, presents many ways for a signal to arrive at the receiver. As a result, throughput may be enhanced through no additional transmit power and little additional processing.

Terrestrial networks often employ single linear vertically polarized (VP) antennas (e.g. cellular mobile, AM and digital radio) or horizontally polarized (HP) antennas (e.g. some FM radio and television broadcast systems). To increase throughput, dual-polarized terrestrial networks may use two linearly polarized (LP) in-phase signals sent from two orthogonal dipoles. The work of Shafi *et al.* [13] uses this arrangement, providing analysis and measurement that consider the impact of elevation angle at the receiver at low gigahertz frequencies. Two common antenna configurations are VP/HP and 45° offset-oriented dipoles. Terrestrial waveforms are not subject to ionospheric depolarization effects such as Faraday rotation [15] and so may typically be received by an aligned dipole arrangement at the receiver.

Circular polarization (CP) may be employed in challenging environments to ensure signal reception. However, this technique may incur additional power transfer loss of up to 50%. At higher frequencies, this is not desirable as linear transmit power is harder to maintain, principally as a result of thermal dissipation issues resulting from smaller surface areas of active devices. In addition, a power transfer maximum is observed in the instance of perfect alignment, or the centre of a FoV, as seen from the perspective of the transmitter. With perfect alignment not typically the case for an immobile receiver, this has negative implications for link capacity in today’s mobile world.

Strongly depolarizing environments, such as the ionosphere, may require dual circular polarization (dual CP) to circumvent depolarizing effects. State-of-the-art satellite systems introduce superposition of left- and right-handed CP signals [10]. As antenna alignment is critical in maintaining performance in a dual CP system, tracking antennas are required. As well as being onerous to install and maintain, mechanical tracking is subject to physical failure.

Deriving full benefit from any of these systems is reliant on a precise alignment of transmit and receive antennas. Performance, including achievable capacity, is greatly affected by misalignment.

Performance of links employing tri-orthogonal antenna configurations has been shown to be less sensitive to orientation and antenna misalignment, providing diversity gain and increased capacity in rich scattering environments [11,12,16]. In effect, the arrangement offers the prospect of orientation robustness over a FoV, on condition that transmitter signalling and receiver processing account for radio wave polarization according to link geometry at the receiver location. At the transmitter, the antenna configuration permits signalling to align with the two dimensions of polarization in the plane perpendicular to the direction of propagation. Any signal component in the direction of propagation is negligible in the far-field. At the receiver, MIMO detection and interference cancellation are feasible due to the tri-orthogonal arrangement. Polarization-time code signalling is practicable [17,18].

In this paper, we propose a three-dimensional channel model incorporating wireless link geometry in addition to polarization mismatch between tri-orthogonal transmit and receive antennas. Simulations are initialized in accordance with reference papers at the FoV centre [11,19]. These papers have been chosen due to, respectively, tri-orthogonal simulation and measurement at the receiver and an analytical approach taken in capacity simulation of LP antennas at millimetre-wave frequencies. Both illustrate results in two-dimensional line graph format. Our model introduces tri-orthogonality at both ends of a link and a three-dimensional approach to analysis. Simulation suggests that tri-orthogonality improves capacity performance in the absence of scattering mechanisms, providing orientation robustness in environments typically encountered at millimetre-wave frequencies. The model is flexible delivering both Rician fading simulations at any operating frequency and link decomposition, a useful technique for evaluating system performance. In addition, the model offers the possibility of introducing near-field and correlation effects while allowing for signalling techniques to take advantage of the geometry of the FoV.

Dipole-to-dipole signal-to-noise (SNR) ratios are calculated, according to which capacity is demonstrated over the FoV. Performance of our tri-orthogonal system is compared and contrasted with both reference papers and with simulated uni- and dual-polarized systems.

Many abstract two-dimensional polarization models for dual-polarized links implicitly assume perfect antenna alignment or apply cross-polar correlations independent of link geometry [10,20]. Capacity improvement through a tri-orthogonal approach in environments both with and without scattering mechanisms is presented through consideration of the three-dimensional geometry and polarization mismatch. This paper presents a useful step in the implementation of a proposed millimetre-wave tri-orthogonal system permitting short-range, high-capacity communication, independent of position and orientation. The paper suggests that the capacity improvement due to a tri-orthogonal approach is available in the absence of a scattering environment.

An allowance built into a system to cater for signal deterioration is known as link margin. A system built solely around an optimal power transfer scenario, typically at the FoV centre, may not propagate a signal, from transmitter to receiver, once power transfer loss in a particular direction exceeds that of the optimal case. A one-size-fits-all approach to link margin results in a system with greater link margin in certain directions than in others. Systems with greater link margin are often more sensitive to signal variation and may frequently suffer the effects of amplifier saturation. A trade-off exists, predominantly at higher frequencies, where linear transmit power becomes more difficult to maintain. Such systems tend to be more expensive as a result. Cost minimization is of considerable benefit.

Implicitly assuming aligned antennas, as is often the case with design, means the effect of polarization mismatch may be inadvertently ignored. In the instance of relative transmitter–receiver movement, this alignment may only occur for a small percentage of the time. Power transfer becomes dependent on the polarization mismatch between transmitter and receiver, itself being a function of relative FoV position. At certain positions in the FoV, this may cause a theoretical infinite loss in power transfer, severely reducing capacity.

This paper aims to demonstrate the effect on capacity over the FoV of power transfer, itself being a function of polarization mismatch, antenna gain and free space path loss in a LoS environment.

## 2. Material and methods

We present the concept of link geometry and its effect on power transfer for a millimetre-wave, tri-orthogonal system. Channels employing MIMO signal propagation have shown improved performance in rich scattering environments [2]. In the absence of a scattering mechanism, diversity gain is greatly reduced. MIMO polarization diversity has demonstrated improved performance over non-polarized diverse MIMO systems in such an environment, this being typically a LoS channel [11,21–23].

To reduce the effects of antenna orientation on the signal, a tri-orthogonal dipole antenna arrangement may be used. Unlike spatially diverse MIMO using three orthogonal polarizations, and systems using dual polarization, tri-orthogonal polarization diverse MIMO permits a compact tri-orthogonal antenna design centred on a single point. Reconfigurable phase-centred radiation patterns are possible, enhancing gain in a given direction, and appropriate for mobile applications as in the IEEE (802.11ad) initiative at 60 GHz.

In the NLoS case, a marked capacity improvement has been observed using three-dimensional polarization diversity over that of dual polarization [11,12]. Tri-orthogonal arrangements introduce a third degree of polarization freedom, the benefit of which may be extended over a FoV. Where deep fading may occur with a uni- or dual-polarized system, it is unlikely that a signal sent from a third orthogonal dipole will suffer the same fading.

Current techniques at low microwave frequencies show a performance improvement, enhanced by multipath effects, but fail to take channel geometry or physical channel effects into consideration [24]. The channel may change rapidly due to relative transmitter–receiver movement. Accurate modelling of such a channel requires inclusion of additional effects such as Doppler phase shift, near-field effects and multipath [2]. Such effects may indeed negate any capacity improvement seen through introduction of a third dipole. An estimated channel model requires a NLoS channel component to be added. Such a model may provide an at-a-glance determination of link performance over the entire FoV. Deep fade areas in the FoV may be determined with subsequent system design taking this into consideration. Power consumption may be used more efficiently so extending battery life, coverage and range. Overall link performance may benefit through enhanced capacity and improved consistency over the FoV.

**Figure 2.**
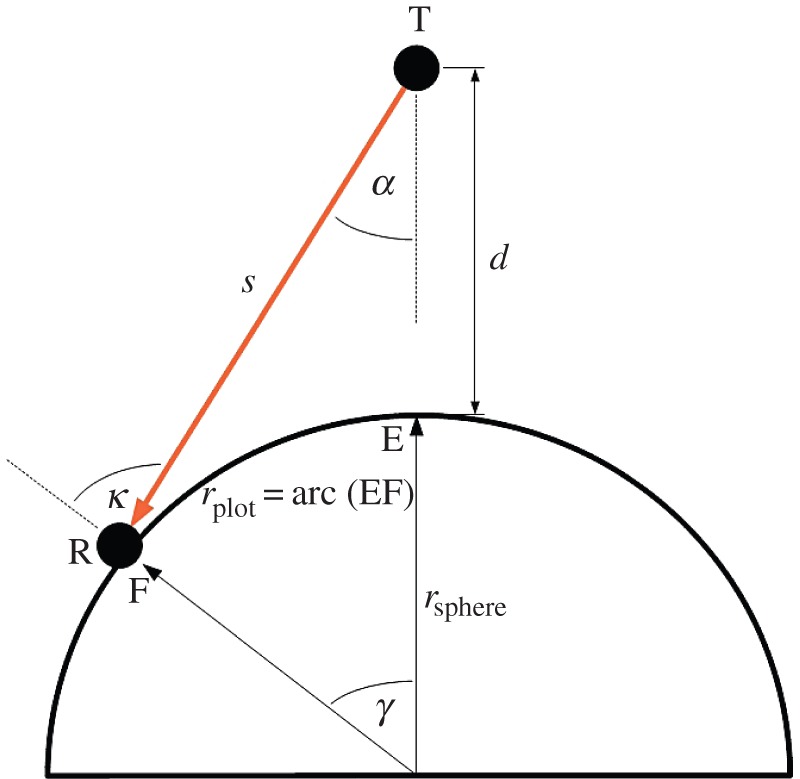
Link geometry: the receiver R is positioned on a semi-circle determined by simple geometry. The proximal distance between T and R is *d*, *s* is path length, while angles *α*, *κ* and *γ* are used to determine relative position. The entire system is rotated about the FoV centre by 360° to develop a spherical surface, forming the FoV. The number of concentric paths on the sphere together with the azimuthal step increment about the FoV centre is set by the user. The algorithm begins at the FoV centre and works out to the circular path where *κ*=90°.

**Figure 3.**
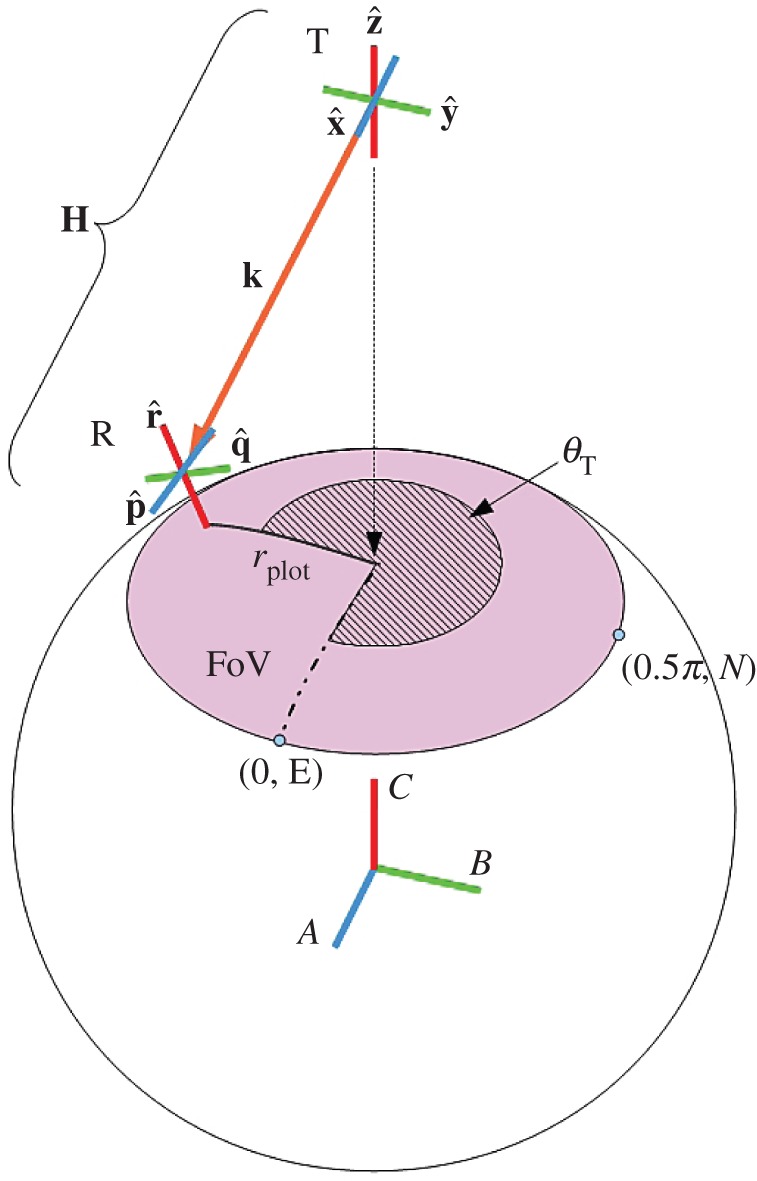
System according to specific location in the FoV: the unit propagation vector $\hat{\text{k}}$ is unique to any position in the FoV and is given according to the azimuthal angle *θ*_T_ and the radial distance from the FoV centre, *r*_*plot*_. Easterly and northerly directions simplify description of the movement of R in the FoV. Unit dipole orientations at R are calculated according to equations (2.14)–(2.16), in three-dimensional space. These, along with the static unit dipole orientations, at T, of $\hat{\text{x}}$, $\hat{\text{y}}$ and $\hat{\text{z}}$, then permit the determination of the parameters required to analyse the channel, **H**, as a function of FoV location.

We assume three mutually orthogonal unit dipole antennas at both transmitter ($\hat{\text{x}}$, $\hat{\text{y}}$, $\hat{\text{z}}$) and receiver ($\hat{\text{p}}$, $\hat{\text{q}}$, $\hat{\text{r}}$). A phase-centred approach at each antenna is required to avoid pattern distortion in the far-field as a result of superposition. All unit dipoles are half-wavelength (λ/2) in length. Without loss of generality, we assume no phase difference between transmitted signals. To understand the benefits of polarization in three dimensions, we look to spherical geometry, which is used in satellite communications design and is given according to textbook definitions [25,26] in figures 2 and 3. The FoV and relevant nomenclature are now introduced. The receiver R is observed to move upon a spherical surface. This surface introduces both variable path length and orientation. The outer radius of the FoV is the point at which unit dipole $\hat{\text{r}}$ is broadside to power transfer from transmitter T. As the FoV is circular, all orientations of the proposed antenna system configuration are included in the FoV. Indeed, the tri-orthogonal system repeats its configuration, and hence the FoV, in six orthogonal directions when the FoV centre is aligned with each of the ± (*A*, *B*, *C*) axes in figure 3.

Referring to figure 3, at T, unit dipole $\hat{\text{x}}$ is aligned with the positive *A* axis, coinciding with an azimuthal angle *θ*_T_, as observed from T, of 0°. Unit dipole $\hat{\text{y}}$ is aligned with the positive *B* axis, coinciding with an azimuthal angle *θ*_T_ of 90°. Unit dipole $\hat{\text{z}}$ is aligned with the positive *C* axis.

At R, and at the FoV centre, unit dipole $\hat{\text{p}}$ is aligned with the positive *A* axis, coinciding with an azimuthal angle *θ*_T_ of 0°. Unit dipole $\hat{\text{q}}$ is aligned with the positive *B* axis, coinciding with an azimuthal angle *θ*_T_ of 90°. Unit dipole $\hat{\text{r}}$ is a radial unit dipole aligned with the positive *C* axis, when R is at the FoV centre.

To effectively explain movement of R within the FoV, easterly and northerly compass directions are invoked, corresponding to azimuthal angles of *θ*_T_ of 0° and 90°, respectively. In figure 3, unit dipole $\hat{\text{p}}$ is deemed to point in an easterly direction, whereas unit dipole $\hat{\text{q}}$ is deemed to point in a northerly direction, for any position of R in the FoV.

Link geometry is determined according to figure 2 and to the following equations:

2.1 $$\begin{array}{rl} \gamma & =\frac{r_{\mathrm{plot}}}{r_{\mathrm{sphere}}}, \end{array}$$

2.2 $$\begin{array}{rl} s & =\sqrt{u^{2}+r_{\mathrm{sphere}}^{2}-2ur_{\mathrm{sphere}}\cos\gamma }, \end{array}$$

2.3 $$\begin{array}{rl} \kappa & =\arcsin\left(\frac{u}{s}\sin\gamma \right), \end{array}$$

2.4 $$\begin{array}{rl} \alpha & =k-\gamma , \end{array}$$

2.5 $$\begin{array}{rl} \gamma_{\max} & =\arccos\left(\frac{r_{\mathrm{sphere}}}{u}\right), \end{array}$$

2.6 $$\begin{array}{rl} s_{\max} & =\sqrt{u^{2}-r_{\mathrm{sphere}}^{2}} \end{array}$$

2.7 $$\begin{array}{rl} \;\text{and}\;\alpha_{\max} & =\arcsin\left(\frac{r_{\mathrm{sphere}}}{u}\right), \end{array}$$

where *u* is *r*_sphere_+*d*.

Observing from T, counterclockwise rotation about the positive *C* axis looking towards the FoV is deemed positive, as in figure 3. Elevation at T is given by *α* with 0° in the negative *C* axis direction, otherwise positive. Elevation at the receiver is given by *κ*, with 0° in the positive *C* axis direction at the FoV centre, otherwise positive. The azimuthal angle *θ*_R_, at R, in the FoV at a position given by the unit propagation vector $\hat{\text{k}}$, differs from the corresponding angle *θ*_T_, at T, by 180°. The receiver R is assumed to be at a distance *s*, from T, that varies according to FoV location. Ideally, power transfer over the FoV is high and constant. The possibility of high capacity communication in any unit vector direction can be evaluated. Where deep fading is encountered, diversity, through redundancy, may be introduced [18,27]. An ideal channel is one where recourse to this is kept to a minimum.

Power transfer between a unit dipole at T and at R is borne out through the Friis formula [28,29]. For a mutually tri-orthogonal antenna transmitter and receiver, nine sub-channel paths are generated. The Friis formula is given as

2.8 $$\frac{P_{R}}{P_{T}}=G_{T}(\phi_{T},\theta_{T})G_{R}(\phi_{R},\theta_{R})(\frac{\lambda }{4\pi s}{)}^{2}e_{\mathrm{pol}}L_{\mathrm{atmos}},$$

where R refers to the receiver, T refers to the transmitter, *P* is power, *G* is dipole gain, λ is transmitted wavelength, *s* is separation of transmitter and receiver, *e*_pol_ is the polarization mismatch between the two dipoles and *L*_atmos_ is atmospheric attenuation due to the interaction with oxygen molecules at 60 GHz and is given here as 15 dB km^−1^ [19].

The power gain *G* of a half-wavelength dipole is given by equation (2.9) [28–30]. This assumes 100% dipole efficiency and is given as

2.9 $$G(\theta ,\phi )=\frac{1.64}{\sin^{2}\phi }\cos^{2}\left(\frac{\pi }{2}\cos\phi \right).$$

In the case of unit dipole $\hat{\text{z}}$ at T, angle *ϕ* is represented by *α*, as shown in figure 2. At T, minimum gain is when *α* is 0°. Maximum gain is when *α* is 90°, which is not in the FoV. We note that the gain of unit dipole $\hat{\text{z}}$ is independent of the azimuthal angle *θ*_T_. For unit dipoles $\hat{\text{x}}$ and $\hat{\text{y}}$, angle *ϕ* can be determined by considering the inner product of the unit dipole vector and the unit propagation vector $\hat{\text{k}}$, where

2.10 $$\hat{\text{k}}=\left[\begin{array}{c} \cos\theta_{T}\sin\alpha \\ \sin\theta_{T}\sin\alpha \\ -\cos\alpha \end{array}\right].$$

**Figure 4.**
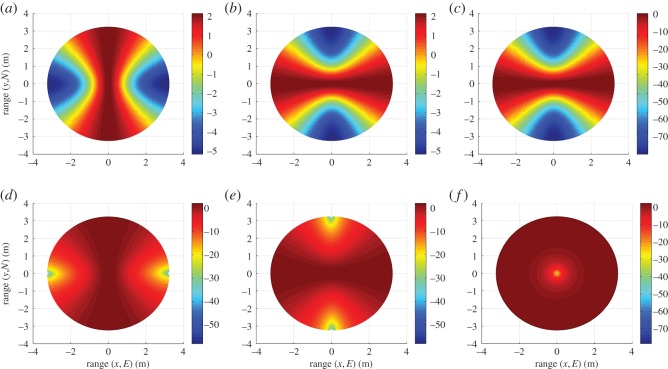
Gain (*G*) (dB) profiles of the six-unit dipoles over the FoV: (*a*) $\hat{\text{x}}$, (*b*) $\hat{\text{y}}$, (*c*) $\hat{\text{z}}$, (*d*) $\hat{\text{p}}$, (*e*) $\hat{\text{q}}$ and (*f*) $\hat{\text{r}}$.

In the case of unit dipole $\hat{\text{r}}$ at R, angle *ϕ* is represented by *κ*, the sum of *α* and *γ*, as in figure 2. For unit dipoles $\hat{\text{p}}$ and $\hat{\text{q}}$, angle *ϕ* is determined in the same manner as that for unit dipoles $\hat{\text{x}}$ and $\hat{\text{y}}$, with *κ* replacing *α*. Figure 4 shows gain profiles of the six unit dipoles.

A signal may incur polarization mismatch loss over a link when two antennas do not have their polarizations perfectly aligned [29,30]. In accordance with the geometry given in figure 2, polarization mismatch *e*_pol_ may be determined for any unit dipole pair. For the unit dipole pair $\hat{\text{z}}\hat{\text{r}}$, the polarization mismatch may be given by the inner product in equation (2.11),

2.11 $$e_{\mathrm{pol}(\hat{\text{z}}\hat{\text{r}})}=|{\hat{\text{z}}}_{⊥\text{k}}\cdot {\hat{\text{r}}}_{⊥\text{k}}{|}^{2},$$

where ${\hat{\text{z}}}_{⊥\text{k}}$ and ${\hat{\text{r}}}_{⊥\text{k}}$ are projections onto the plane perpendicular to the unit propagation vector $\hat{\text{k}}$. As $\hat{\text{r}}$ is a radial dipole, while $\hat{\text{z}}$ is a static dipole in the zenith direction, no polarization mismatch occurs for this unit dipole pair.

The projection of an arbitrary vector **v** onto the plane perpendicular to $\hat{\text{k}}$ may be given by

2.12 $${\text{v}}_{⊥\text{k}}=({\text{I}}_{3}-\hat{\text{k}}{\hat{\text{k}}}^{T})\hat{\text{v}},$$

which can then be normalized to give,

2.13 $${\hat{\text{v}}}_{⊥\text{k}}=\frac{{\text{v}}_{⊥\text{k}}}{|{\text{v}}_{⊥\text{k}}|}.$$

**Figure 5.**
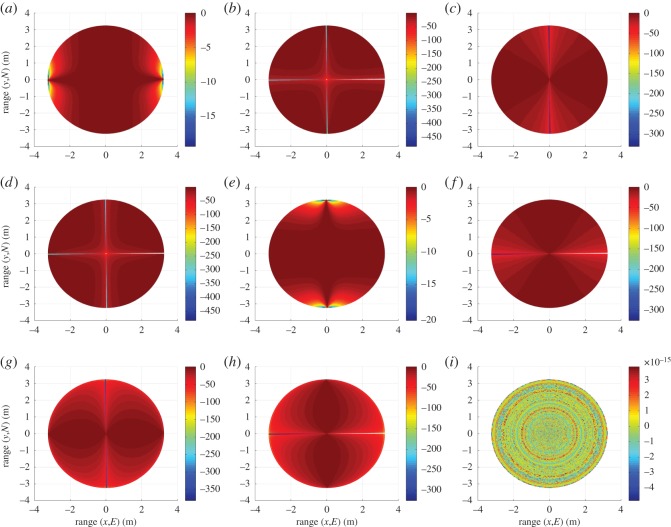
Polarization mismatch (*e*_pol_) (dB) profiles for each unit dipole pair: (*a*) $\hat{\text{x}}\hat{\text{p}}$, (*b*) $\hat{\text{y}}\hat{\text{p}}$, (*c*) $\hat{\text{z}}\hat{\text{p}}$, (*d*) $\hat{\text{x}}\hat{\text{q}}$, (*e*) $\hat{\text{y}}\hat{\text{q}}$, (*f*) $\hat{\text{z}}\hat{\text{q}}$, (*g*) $\hat{\text{x}}\hat{\text{r}}$, (*h*) $\hat{\text{y}}\hat{\text{r}}$ and (*i*) $\hat{\text{z}}\hat{\text{r}}$.

Polarization mismatch profiles are given in figure 5 for all unit dipole pair combinations.

To describe orientation of the six dipoles, a set of right-handed Cartesian axes is invoked, as per figure 3. Axes *A*, *B* and *C* are used to describe receiver position on the sphere surface. Lengths *a*, *b* and *c* are along these axes, respectively, and are normalized by *r*_sphere_. The unit vectors representing unit dipoles $\hat{\text{x}}$, $\hat{\text{y}}$, $\hat{\text{z}}$ and $\hat{\text{r}}$, are, respectively, $\hat{\text{x}}={\left[1\;0\;0\right]}^{T}$, $\hat{\text{y}}={\left[0\;1\;0\right]}^{T}$, $\hat{\text{z}}={\left[0\;0\;1\right]}^{T}$ and

2.14 $$\hat{\text{r}}=\left[\begin{array}{c} \cos\theta_{T}\sin\gamma \\ \sin\theta_{T}\sin\gamma \\ \cos\gamma \end{array}\right],$$

where the superscript T denotes transpose.

The position on the spherical surface, relative to the FoV centre position, must be known to describe the orientation of dipoles *p* and *q*,

2.15 $$\hat{\text{p}}=\left[\begin{array}{c} \cos({\mathrm{atan}}_{2}(a,c))\\ 0\\ -\sin({\mathrm{atan}}_{2}(a,c)) \end{array}\right]$$

and

2.16 $$\hat{\text{q}}=\left[\begin{array}{c} -\sin(\arcsin(b))\sin({\mathrm{atan}}_{2}(a,c))\\ \cos(\arcsin(b))\\ -\sin(\arcsin(b))\cos({\mathrm{atan}}_{2}(a,c)) \end{array}\right],$$

where atan_2_(*a*,*c*) is described as

2.17 $${\mathrm{atan}}_{2}(a,c)=\left\{ \begin{array}{ll} \arctan\left(\frac{a}{c}\right) & \left(c≻0\right)\\ \arctan\left(\frac{a}{c}\right)+\pi & \left(a⪰0,c≺0\right)\\ \arctan\left(\frac{a}{c}\right)-\pi & \left(a≺0,c≺0\right)\\ +\frac{\pi }{2} & \left(a≻0,c=0\right)\\ -\frac{\pi }{2} & \left(a≺0,c=0\right)\\ \mathrm{undefined} & (a=0,\;c=0). \end{array}\right.$$

At the FoV centre in figure 3, *a*=0, *b*=0 and *c*=1.

Received symbols may be given according to the textbook definition [2] and equation (2.21), where

2.18 Y=HX+N.

In equation (2.21), **Y** is the set of received signals at the receiver, **H** represents a 3×3 complex fading channel matrix, **X** is a block of symbols sent and **N** is complex additive white Gaussian noise (AWGN) at the receiver R.

The complex fading channel matrix may be decomposed into the sum of a deterministic average LoS component ($\bar{\text{H}}$), determined by application of equation (2.8) for each subchannel, and a stochastic variable scattered NLoS component ($\tilde{\text{H}}$) given by,

2.19 $$\text{H}=\sqrt{\frac{K}{1+K}}\bar{\text{H}}+\sqrt{\frac{1}{1+K}}\tilde{\text{H},}$$

where *K* is the Ricean *K*-factor [2]. Note that *K*=0 corresponds to a pure stochastic Rayleigh, or complex Gaussian, fading channel with no LoS component, while K=∞ corresponds to a pure AWGN fading channel or LoS system, with no signal reflections in the channel. By adding a complex Gaussian component, we introduce multipath, providing enhanced capacity performance over the channel through reflections.

For this paper, we simulate channel capacity at an instant in time for Ricean *K*-factors of 0 and ∞. Without loss of generality, we assume the phase arguments of the deterministic LoS coefficients in $\bar{\text{H}}$ to be zero. We also assume all dipoles at T to be co-located, and the same assumption is made for dipoles at R. The LoS power transfer for each unit dipole pair, given by equation (2.8), forms a basis to determine the nine channel coefficients in equation (2.20), unique to a FoV location. These coefficients are obtained from $\sqrt{P_{R}/P_{T}}$ and may be considered as being proportional to signal voltage amplitude changes as a result of channel propagation. The polarization mismatch factor, *e*_pol_, includes projections via equation (2.12), with the 3×3 matrix **H** being at most rank 2 in the LoS case. Channel rank, and therefore capacity, may be increased via multipath in the Rayleigh fading channel case.

Applications of the channel model, in the AWGN fading or LoS case, are currently restricted and may include an improved determination of the LoS component used for channel state information (CSI) to optimize transmission. Use of omnidirectional antennas enhances multipath in the case of a Rayleigh fading channel, increasing capacity over the FoV. Doppler frequency shift, caused by relative transmitter–receiver motion, is omitted, as are near-field and correlation effects. A channel matrix **H** for each position in the FoV may be determined according to the calculation of the nine subchannels using equation (2.8). For M receiver elements, or dipoles in this instance, and N transmitter elements, or dipoles in this instance, the channel matrix is of the form given below,

2.20 $$\text{H}=\left[\begin{array}{ccc} h_{\hat{\text{p}}\hat{\text{x}}} & h_{\hat{\text{p}}\hat{\text{y}}} & h_{\hat{\text{p}}\hat{\text{z}}}\\ h_{\hat{\text{q}}\hat{\text{x}}} & h_{\hat{\text{q}}\hat{\text{y}}} & h_{\hat{\text{q}}\hat{\text{z}}}\\ h_{\hat{\text{r}}\hat{\text{x}}} & h_{\hat{\text{r}}\hat{\text{y}}} & h_{\hat{\text{r}}\hat{\text{z}}} \end{array}\right],$$

where the matrix coefficients in equation (2.20) represent signal transfer between a unit dipole pair.

Capacity for M receivers and N transmitters is given according to equation (2.21) [17,18,31] as

2.21 $$C=\log_{2}\left|({\text{I}}_{\text{M}}+\frac{\rho }{N}\text{H}{\text{H}}^{†})\right|,$$

where **I** represents an identity matrix, *ρ* the average SNR and ^†^ denotes the Hermitian transpose. The SNR divided by the number of transmitter elements N ensures that a like-to-like comparison is made for all systems. With identical transmit power assumed in all cases, a 3×3 system spreads transmit power over the FoV to provide orientation robustness, rather than concentrating power at the FoV centre.

## 3. Results

**Figure 6.**
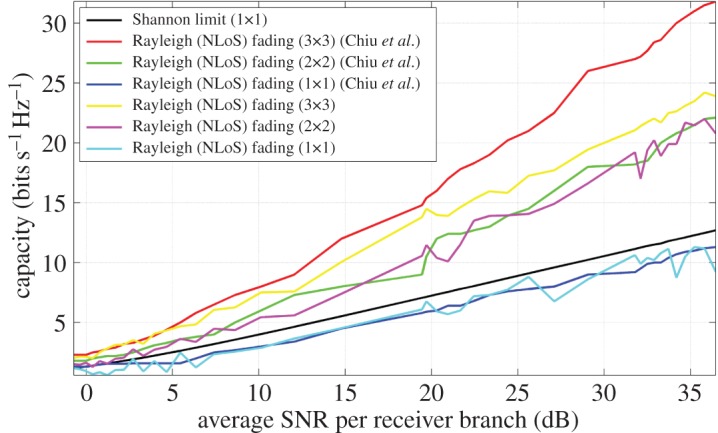
Simulated capacity of uni-polarized, dual-polarized and tri-orthogonal arrangements at 2.55 GHz, in a Rayleigh (NLoS) fading channel, and as a function of average SNR per receiver branch at the FoV centre, according to prior work [11]. A Ricean factor *K* of 10^−2^ is used. The transmitter is perfectly aligned with the receiver along the positive *C* axis, as in figure 3. The difference in capacity between the tri-orthogonal, or 3×3, arrangement of the presented model and the simulated and measured 3×3 arrangement in the work of Chiu *et al.* is due to a non-orthogonal arrangement at the transmitter being previously employed.

In order to simulate, comparison with prior work in the field was invoked [11,19]. Figure 6 shows data taken at the FoV centre, at 2.55 GHz in a Rayleigh fading (NLoS) channel [32], with T and R aligned along the positive *C* axis as per figure 3, for a range of average SNR per receiver branch values [11]. As expected, both the uni-polarized system developed in the model, as well as that of Wang *et al.* [19], approach the Shannon capacity limit due to perfect alignment, thus optimal signal transfer, along this axis. The same analysis applies to the dual-polarized case. A discrepancy exists between that of the simulated tri-orthogonal NLoS, or 3×3, case presented in this paper and that of prior work [11]. This is due only to the receiver being a tri-orthogonal arrangement, the transmitter being a threefold, LP system. As a consequence, the arrangement demonstrates a threefold increase in capacity over that of a uni-polarized arrangement. In the presented channel model, this is not the case due to the tri-orthogonal arrangement at T and link geometry. In figure 6, this difference in 3×3 capacity is clearly observed.

**Figure 7.**
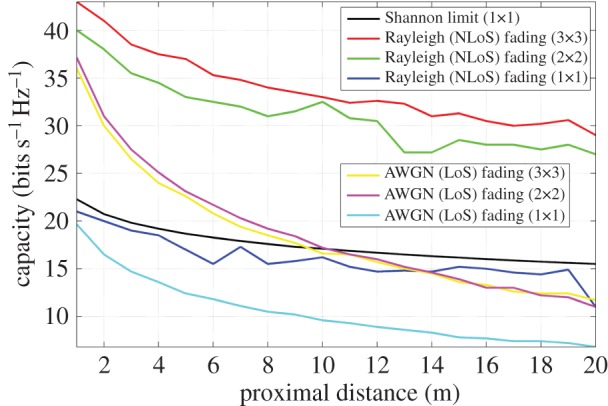
Simulated capacity at the FoV centre of uni-polarized, dual-polarized and tri-orthogonal arrangements at 60 GHz, in both AWGN (LoS) and Rayleigh (NLoS) fading channels, and as a function of proximal distance, or *d* as shown in figure 2. Ricean factors of 10^−2^ and 10^3^ are used to simulate Rayleigh and AWGN fading channels, respectively. The transmitter is perfectly aligned with the receiver along the positive *C* axis, as in figure 3. The dual-polarized and tri-orthogonal arrangements are extensions, through analysis of link geometry, to the uni-polarized system previously presented [19].

Figure 7 illustrates a uni-polarized LoS capacity comparison, at 60 GHz, of the channel model with that of previous work in this area [19]. This is extended to both dual-polarized and tri-orthogonal systems, since all these arrangements base their channel coefficients on an average SNR at a point in the FoV, discerned through link geometry. The average SNR per receiver branch is calculated over a proximal distance, *d*, ranging from 1 to 20 m. The uni-polarized LoS capacity presented in this channel model shows good agreement with the line corresponding to the channel exponent of *n*=1.55 in fig. 3 of Wang *et al* [19].

The channel model is inherently based on SNR, as is any determination of link performance. Comparison with prior work [11,19] allows for the channel model to be compared over a full range of variables. These include frequency, power, NLoS and LoS configuration, and degrees of polarization.

**Figure 8.**
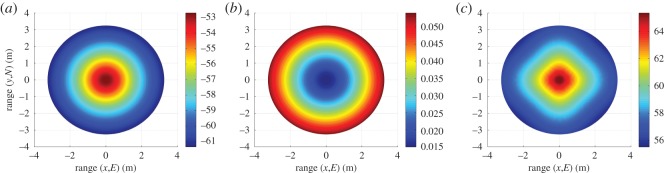
(*a*) Free space path loss (dB), (*b*) atmospheric loss *L*_atmos_ (dB) and (*c*) average SNR per receiver branch for a tri-orthogonal system (dB) profiles over the FoV.

We present simulated three-dimensional results over a FoV using an operating frequency of 60 GHz, a transmit power of 40 dBm, a bandwidth of 7 GHz and a system noise temperature [19] of 290 K . The propagation distance at the FoV centre is 1 m, corresponding to a close proximity wireless personal area network (WPAN), while a spherical radius of 6 m is employed. This corresponds to a maximum average SNR per receiver branch at the FoV centre of 65 dB, as shown in figure 8*c*. Ricean *K*-factors of *K*=0 and K=∞, corresponding to pure Rayleigh [19,33] and AWGN fading channels, are used in these simulations. System-specific values of free space path loss, atmospheric loss and SNR are illustrated in figure 8.

**Figure 9.**
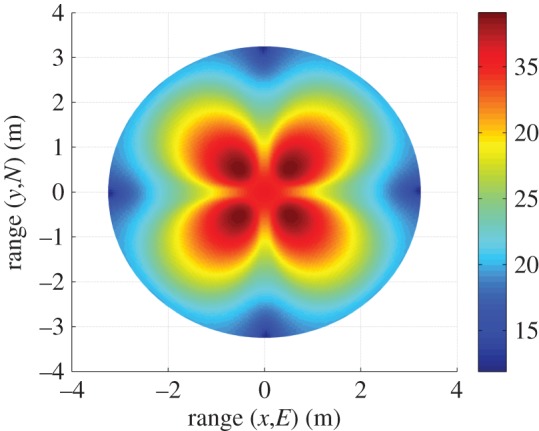
Tri-orthogonal, or 3×3, AWGN fading channel capacity (bits s^−1^ Hz^−1^).

**Figure 10.**
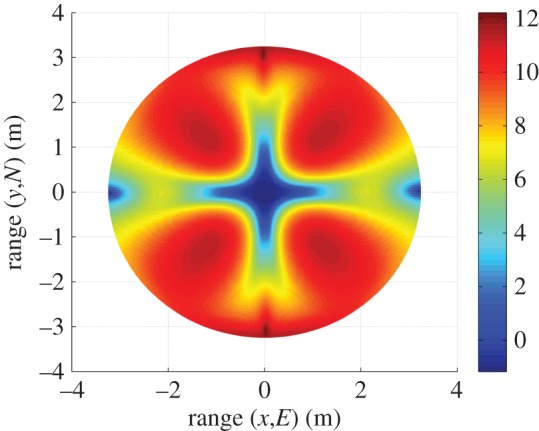
Capacity advantage (bits s^−1^ Hz^−1^) of a tri-orthogonal, or 3×3, system over that of a dual-polarized, or 2×2, system in an AWGN fading channel.

**Figure 11.**
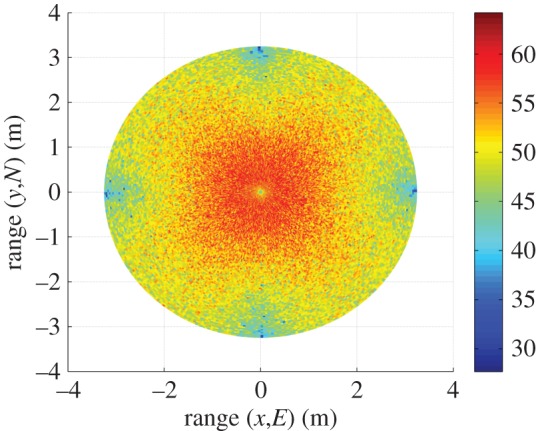
Tri-orthogonal, or 3×3, Rayleigh fading channel capacity (bits s^−1^ Hz^−1^).

**Figure 12.**
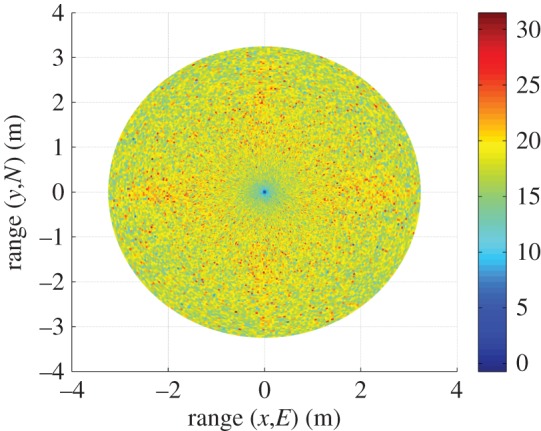
Capacity advantage (bits s^−1^ Hz^−1^) of a tri-orthogonal, or 3×3, system over that of a dual-polarized, or 2×2, system in a Rayleigh fading channel.

**Figure 13.**
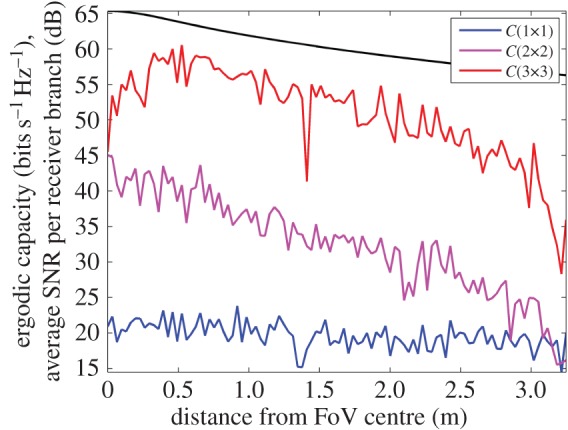
Rayleigh fading channel capacity (bits s^−1^ Hz^−1^) profile at *θ*_T_=0° for uni-polarized, dual-polarized and tri-orthogonal systems. The average SNR per receiver branch as a function of distance from the FoV centre is also shown.

**Figure 14.**
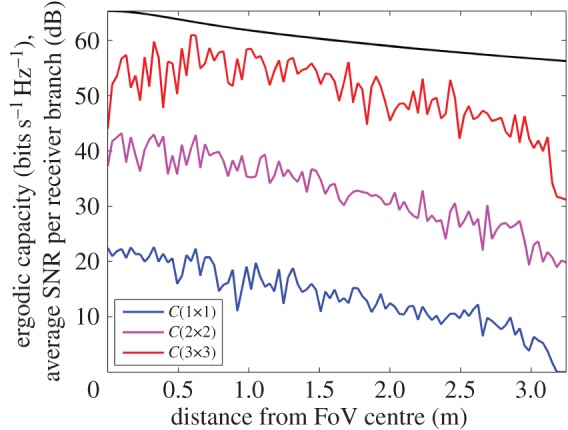
Rayleigh fading channel capacity (bits s^−1^ Hz^−1^) profile at *θ*_T_=90° for uni-polarized, dual-polarized and tri-orthogonal systems. The average SNR per receiver branch as a function of distance from the FoV centre is also shown.

**Figure 15.**
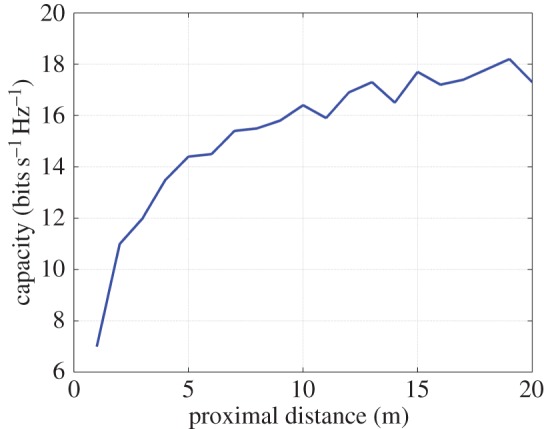
Capacity advantage (bits s^−1^ Hz^−1^) of a rich scattering environment such as a Rayleigh fading channel over that of an AWGN fading channel. The proximal distance is shown in figure 2 as *d*.

Figure 9 shows the AWGN fading channel capacity over the FoV, as produced by the tri-orthogonal 3×3 system. Figure 10 shows the AWGN fading channel capacity advantage over the FoV, as produced by a tri-orthogonal 3×3 system over a dual-polarized 2×2 system. Figure 11 shows the Rayleigh fading channel capacity over the FoV, as produced by the tri-orthogonal 3×3 system. Figure 12 shows the Rayleigh fading channel capacity advantage over the FoV, as produced by a 3×3 system over a 2×2 system. We note from these figures that:

— the 3×3 system in both AWGN and Rayleigh fading regimes exhibits a channel capacity higher than that of the 2×2 and uni-polarized 1×1 systems over the majority of the FoV. This is most clearly seen in figures 9 and 10. An absence of a scattering mechanism at millimetre-wave frequencies does not stop the inclusion of a third orthogonal dipole from improving performance over the majority of the FoV. The capacity is more consistent over the FoV and is observed to approach the Shannon capacity limit in the instance of a Rayleigh fading channel. This is seen in figures 11 and 12;
— at the FoV centre, the 3×3 system experiences a small capacity disadvantage when compared to the 2×2 system, as seen in figures 10 and 12. This is due to redundancy of the third orthogonal unit dipole pair, or $\hat{\text{z}}\hat{\text{r}}$, at this point and is most prominent in the AWGN channel. In an absence of a scattering mechanism, any propagating signal from unit dipole $\hat{\text{z}}$ cannot be reflected into this region, nor can unit dipole $\hat{\text{r}}$ receive signals that are incumbent upon it end on. As the 2×2 system exhibits an approximate doubling of capacity over that of the 1×1 system and the FoV centre is where the 2×2 capacity is at a maximum, this small capacity disadvantage is seen as a reasonable trade-off to make for increased throughput and consistency over the majority of the FoV, known as orientation robustness. In the Rayleigh fading channel, this disadvantage is mitigated by the increased probability of propagation between two perfectly misaligned unit dipoles in a rich scattering environment. It is of note that in figure 7, the capacity disadvantage is not seen for the 3×3 system. This is attributable to an average of capacity being obtained from orthogonal azimuthal angle positions incrementally close to the exact FoV centre. Considering the orthogonal azimuthmal angle positions in figures 13 and 14, we observe that, although the dip in capacity for the 3×3 system is prevalent in both instances, capacity does not fall below that of the 2×2 system;
— capacity for the 3×3 system in an AWGN channel is seen to be highest at four offset positions approximately 10° off-centre and at 45°, 135°, 215° and 305° azimuth. This is most clearly observed in figure 9. At these points, a tripling of capacity compared to the 1×1 system, with capacity approaching the 3×3 Shannon capacity limit, is observed. This is suggestive of orientation robustness even in the absence of a scattering mechanism, this environment being typically prevalent at millimetre-wave frequencies;
— in a Rayleigh fading channel, capacity is observed to increase through inclusion of a third orthogonal unit dipole over the majority of the FoV. This is observed in figure 12. An approximate tripling of capacity, with reference to the 1×1 case, is observed for all positions around the FoV centre, with capacity approaching the 3×3 Shannon capacity limit in figure 11. This high capacity zone is more pronounced than for the AWGN channel, this being due to the increased probability of propagation between two perfectly misaligned dipoles in a rich scattering environment. The capacity disadvantage at the FoV centre, observed in figures 11 and 12, is once again observed in figures 13 and 14, at azimuthal angles of *θ*_T_ of 0° and 90°, respectively; and
— the channel capacity advantage at the FoV centre, as observed in a Rayleigh fading channel, when compared to an AWGN fading channel, increases with increasing path distance, or decreasing average SNR per receiver branch, before levelling out. This is shown in figure 15. The rich scattering environment is observed to be more beneficial for lower average SNR per receiver branch values.

## 4. Discussion

The analysis provided in this paper suggests that orientation robustness and overall increased capacity performance for communication at 60 GHz [34–36] is provided over the majority of a FoV by inclusion of a third orthogonal dipole at both transmitter and receiver. At millimetre-wave frequencies, and in the extreme case of an absence of channel scattering mechanism, multipath effects do not assist propagation. At lower microwave frequencies, the advantage of a rich scattering environment is often included in reference papers that consider the benefits of MIMO operation. Analysis is often limited to perfect alignment, or a few specific orientations. The presented paper’s simulations suggest that the third dipole provides orientation robustness and improved capacity performance, even in the absence of a channel scattering mechanism. A small capacity disadvantage is noted at the FoV centre, where the 2×2 system provides optimal signal transfer, but is seen as a permissible trade-off for improved orientation robustness and overall capacity performance.

The simulated results presented here are based on an analysis of link geometry in three dimensions, together with comparative modelling at a FoV centre, using uni-polarized, dual-polarized and tri-orthogonal arrangements, and two sets of operational parameters. Good agreement is found, through this comparison, with both simulated and measured results from previous papers.

With a phase-centred design avoiding pattern distortion in the far-field, and through the application of phased feeding techniques, beamsteering of antenna radiation patterns becomes possible. The capacity over the FoV may in effect be adjusted through superposition of individual dipole radiation patterns, resulting in a maximum at a specific FoV location where the receiver exists. In addition, matrices may be added to the existing channel matrix that describe near-field antenna effects, such as correlation and mutual coupling effects. In this paper, a fundamental approach highlighting the importance of polarization diversity is assumed. As a consequence, we do not apply phased feeding techniques and assume no correlation effects and no mutual coupling between dipoles, due to a tri-orthogonal arrangement [37]. The model does not take relative transmitter–receiver velocity into account, but rather demonstrates the effect of antenna misalignment on system performance, and a method of mitigating against it.

In a typical environment at millimetre-wave frequencies, applications of orientation robustness through tri-orthogonality range from very short-range personal communications, such as WPAN devices, to implementation of the IEEE (802.11ad) initiative at 60 GHz, an integral part of a tri-band wireless communications solution.
